# In Vitro Analysis of the Fracture Resistance of CAD/CAM Denture Base Resins

**DOI:** 10.3390/ma11030401

**Published:** 2018-03-08

**Authors:** Otto Steinmassl, Vincent Offermanns, Wolfgang Stöckl, Herbert Dumfahrt, Ingrid Grunert, Patricia-Anca Steinmassl

**Affiliations:** 1 University Hospital for Cranio-Maxillofacial and Oral Surgery, Department of Dental and Oral Medicine and Cranio-Maxillofacial and Oral Surgery, Medical University of Innsbruck, Anichstr. 35, A-6020 Innsbruck, Austria; otto.steinmassl@i-med.ac.at (O.S.); vincent.offermanns@i-med.ac.at (V.O.); 2 University Hospital for Dental Prosthetics and Restorative Dentistry, Department of Dental and Oral Medicine and Cranio-Maxillofacial and Oral Surgery, Medical University of Innsbruck, Anichstr. 35, A-6020 Innsbruck, Austria; wolfgang.stoeckl@i-med.ac.at (W.S.); herbert.dumfahrt@i-med.ac.at (H.D.); ingrid.grunert@i-med.ac.at (I.G.)

**Keywords:** CAD/CAM dentures, complete dentures, dental materials, PMMA resin, breaking load, fracture toughness, elastic modulus

## Abstract

Computer-aided design and computer-aided manufacturing (CAD/CAM) denture base manufacturers claim to produce their resin pucks under high heat and pressure. Therefore, CAD/CAM dentures are assumed to have enhanced mechanical properties and, as a result, are often produced with lower denture base thicknesses than conventional, manually fabricated dentures. The aim of this study was to investigate if commercially available CAD/CAM denture base resins have more favourable mechanical properties than conventionally processed denture base resins. For this purpose, a series of three-point bending tests conforming to ISO specifications were performed on a total of 80 standardised, rectangular CAD/CAM denture base resin specimens from five different manufacturers (AvaDent, Baltic Denture System, Vita VIONIC, Whole You Nexteeth, and Wieland Digital Dentures). A heat-polymerising resin and an autopolymerising resin served as the control groups. The breaking load, fracture toughness, and the elastic modulus were assessed. Additionally, the fracture surface roughness and texture were investigated. Only one CAD/CAM resin showed a significantly increased breaking load. Two CAD/CAM resins had a significantly higher fracture toughness than the control groups, and all CAD/CAM resins had higher elastic moduli than the controls. Our results indicate that CAD/CAM denture base resins do not generally have better mechanical properties than manually processed resins. Therefore, the lower minimum denture base thicknesses should be regarded with some caution.

## 1. Introduction

Cracks and fractures are the most common causes of failure among removable complete dentures [1]. The University Hospital for Dental Prosthetics and Restorative Dentistry at the Medical University of Innsbruck reports around 175 incidents of denture cracks and fractures per year. Besides poor denture design [2], denture failure is attributed to the denture base resins’ poor mechanical properties [3]. For enhancing these properties, different targets have been pursued, such as altering the microstructure by admixing additives [4,5,6], increasing the liquid/powder ratio [7], and optimising the processing protocol [8,9]. A comparative study from 2012 concluded that the introduction of completely new manufacturing techniques might be necessary [4].

The recently introduced option of computer-aided design and computer-aided manufactured (CAD/CAM) removable dentures [10,11,12,13,14] definitely addresses this demand, as it has fundamentally changed the manufacturing process. Instead of manually mixing the resin powder and liquid and then submitting the immersion to an arbitrarily chosen curing protocol, the poly(methyl methacrylate) (PMMA) resin blocks for CAD/CAM denture bases are industrially fabricated [15,16] and cured under “great heat and pressure” [17,18]. Therefore, it has been assumed that the CAD/CAM denture base resins are highly condensed and have fewer micro-porosities [17]. This, in consequence, would mean that CAD/CAM denture base resins could have superior mechanical properties [8], which is probably why some of the CAD/CAM denture manufacturers advertise that their products have a very low minimum material thickness [19].

It was the aim of this study to investigate if different commercially available CAD/CAM denture base resins have in fact better mechanical properties than conventionally processed self- or heat-curing resins (*H*_0_-hypothesis: CAD/CAM denture base resins do not have a different breaking load, fracture toughness, or elastic modulus than heat- or self-curing resins).

In preliminary experiments, the different surface textures of the fracture fragments attracted attention. While some specimens had smooth, almost glass-like fracture surfaces, other specimens showed macroscopically rough fracture surfaces. Therefore, it became a further aim to assess if the fracture surface roughness differs significantly between CAD/CAM-fabricated and conventionally manufactured denture base resins (*H*_0_-hypothesis: CAD/CAM denture base resins do not have a different fracture surface roughness than heat- or self-curing resin). In addition, potential statistical correlations between the fracture surface roughness and the mechanical behaviour were investigated (*H*_0_-hypothesis: There is no statistical correlation between breaking load, fracture toughness, or elastic modulus and the fracture area’s surface roughness).

## 2. Materials and Methods

### 2.1. Study Specimens

In the present in vitro study, a total of 80 rectangular denture base resin samples, sized 39 mm × 8 mm × 4 mm, were examined. These samples were divided into eight groups (six study groups and two control groups) with 10 specimens each. 

The specimens for study groups 1 to 4 were provided by different CAD/CAM denture base resin manufacturers: AvaDent (AD), Baltic Denture System (BDS), Vita VIONIC (VV), and Wieland Digital Dentures (WDD). Since Whole You customarily applies a light-curing topcoat to all their dentures, the manufacturer was asked to provide two different types of specimens (study groups 5 and 6). The specimens of study group 5 were prepared without the customary full surface coating (WNu) and the specimens of study group 6 were prepared with the regular coating (WNc). All the CAD/CAM specimens were milled by the manufacturers, from their respective CAD/CAM denture resin pucks. 

The specimens for the control groups were made from two different conventionally processed denture base resins. Control group 1 (C1) represented the standard method used at the Medical University of Innsbruck, and the specimens were made from heat-curing resin (Candulor Aesthetic Red) using the compressed mould technique. The moulds were made from class IV gypsum (SheraPure, SHERA Werkstoff-Technologie GmbH & Co. KG, Lemförde, Germany) and isolated with plaster-against-resin separating liquid (Separating Fluid, Ivoclar Vivadent, Schaan, Liechtenstein). The resin was prepared according to the manufacturer’s instructions, using a long-term heat polymerisation cycle (a 75 °C water bath for 8.5 h at a pressure of 3.5 bar). The specimens for control group 2 (C2) were made from self-curing resin (Candulor Aesthetic Blue) using the pouring technique. The moulds for control group 2 were made from addition-cured silicone for duplication (Brasil 22, Dentona Dortmund, Germany). The resin was again prepared according to the manufacturer’s instructions, and the polymerisation cycle followed a short-term water bath polymerization at a temperature of 40 °C for 15 min at a pressure of 2.0 bar. The surfaces of the C1 and C2 specimens were then wet-ground with metallographic grinding papers with a grain size of 18 μm, to obtain smooth and flat surfaces. All tested groups and their manufacturers are listed in detail in Table 1.

Before being submitted to analysis, all specimens were stored in 100 mL of water for 7 days at a controlled temperature of 37 °C in darkness. After this immersion period, the specimens were placed in a new container with water at a temperature of 23 °C for 1 h prior to testing, then removed and towel-dried with a paper tissue.

### 2.2. Three-Point Bending Test

**Figure 1:**
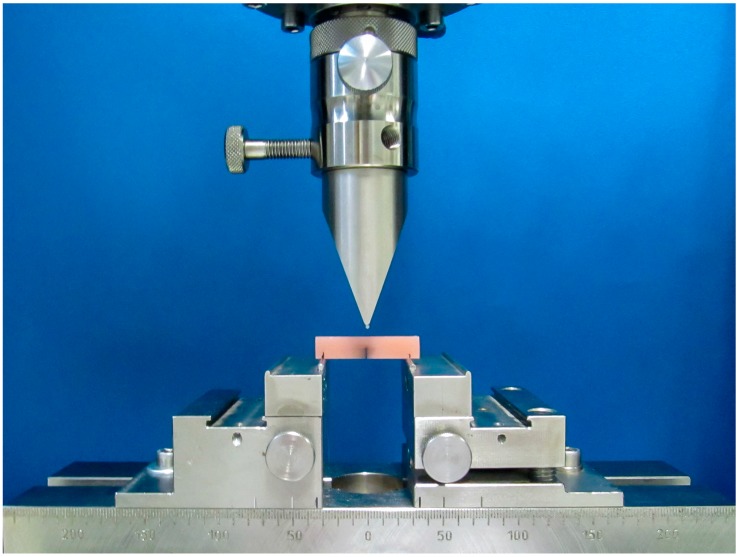
Experimental setup for the three-point bending test.

A series of three-point bending tests were performed on all specimens (Figure 1), following the ISO 20795-1:2013 [20] in a climate-controlled environment. The ISO required the standardised application of a pre-crack in the middle of each specimen. For this purpose, the specimens were cut to a standardised depth of 3.0 mm using a disc saw with a diamond saw blade thickness of 0.5 mm under constant air cooling. The pre-cracks were then further extended, as demanded by the ISO requirements, by performing scalpel cuts between 100 μm and 400 μm in depth, applied with constant pressure. The cutting blade was replaced after five cuts. Before beginning the three-point bending test, the pre-crack regions were demarcated with black ink.

The fracture tests were performed using a universal material testing machine (Zwick/Roell Z010, Zwick GmbH & Co. KG, Ulm, Germany), controlled via TestXpert-V9.0 software (Zwick GmbH & Co. KG, Ulm, Germany). Again, following the ISO requirements, the testing machine was programmed to a constant displacement rate of 1 mm/min, a pre-load of 5.0 N, and a pre-load speed of 10 mm/min. The test was considered finished when the current load was reduced to 5% of the maximum load, or was less than 1.0 N [20]. Counterforts measuring 2.5 mm in diameter were laser-fixed onto the positioner at a distance of 32.0 mm. The plunger was modified by laser-fixing a counterfort measuring 1.5 mm in diameter. The specimens were then centred on the positioner, so that the pre-crack was on the bottom side of the specimen. Following the aforementioned protocol, increasing pressure was applied until the material fractured, and the load/deflection curve was registered, together with the maximally applied force, which represented the breaking load ($F_{max}$). To ensure the quality of the obtained data, preliminary bending tests were performed by two different examiners using two different material testing machines of the same kind. Since there were no significant differences, the results were considered to be reproducible.

All fracture surfaces were inspected using an incident light microscope (SteREO Lumar.V12, Carl Zeiss AG, Oberkochen, Germany) at a 42-fold magnification and a field of vision of 5.5 mm. After ensuring that the fracture surfaces were free of accidental macro-pores, the pre-crack dimensions were calculated by dividing the fracture area into quarters and calculating the mean of the pre-crack lengths from the three-quarter lines, as required by the ISO specifications. The fracture toughness (*K_Ic_*) was calculated using the following formulas [20]:

(1) $$K_{Ic}=\frac{f\left(x\right)\cdot F_{max}\cdot l_{t}}{b_{t}\cdot h_{t}{}^{\frac{3}{2}}}\cdot \sqrt{10^{-3}}\;\mathrm{MPa}\cdot m^{1/2}$$

(2) $$f\left(x\right)=\frac{3x^{\frac{1}{2}}\left[1.99-x\left(1-x\right)\left(2.15-3.93x+2.7x^{2}\right)\right]}{2\left(1+2x\right){\left(1-x\right)}^{\frac{3}{2}}}$$

(3) $$x=\frac{a}{h_{t}}$$

In these equations, $F_{max}$ was the maximally applied force (N), $l_{t}$ was the span (mm) between the counterforts, $h_{t}$, and $b_{t}$ were the height (mm) and width (mm) of the specimen, and a was the length (mm) of the pre-crack. The fracture toughness describes the ability of a specimen containing a predetermined breaking point to resist failure. 

In addition, the elastic modulus *E*, a measure for the material stiffness, was calculated using the formula

(4) $$E=\frac{F_{max}\cdot l_{t}{}^{3}}{4\cdot h^{3}\cdot b\cdot s}\;\mathrm{MPa}$$

in which h and b were the height (mm) and the width (mm) of the specimen’s fracture surface, and s was the plunger displacement (mm) at maximally applied force.

### 2.3. Fracture Surface Roughness Measurements and Fracture Surface Texture

After the mechanical data were obtained, the roughness of the fracture areas (*R_a_*) of all AD, BDS, VV, WDD, C1, and C2 specimens was measured using a contact profilometer (Taylor Hobson Form TalySurf Serie 2 FTS S2, Leicester, UK). Since WNc and WNu both used the same resin, the fracture surface measurements were performed only on the WNc group, but the results may be considered to apply to both WN groups. The roughness parameter *R_a_* stands for “roughness average”. It is the most common parameter for describing a surface’s roughness and is defined as the arithmetic mean of the absolute values of the roughness profile ordinates. The measurements were performed perpendicular to the surface. The profilometer filter settings were chosen according to the respective ISO norm [21]. Three standardised measurements of 4.0 mm were performed. The cut-off wavelength was set at λ = 0.8 mm, meaning that roughness peaks larger than 0.8 mm did not contribute to the result. 

To gain a better understanding of the specific fracture surface configurations, scanning electron microscope (SEM) images were produced using a LEO-1550 FE-SEM (Carl Zeiss GmbH, Vienna, Austria) at magnifications of 100-fold and 450-fold.

### 2.4. Statistics

The data were handled using SPSS Statistics 22 (IBM, Armonk, NY, USA) and R 3.4.1 (R Foundation for statistical computing, Vienna, Austria). For the statistical analysis, the data were assessed by the inspection of box plots regarding outliers. The Shapiro-Wilk’s test and QQ-plots were used to test the data’s normal distribution. Means and standard deviations (SD) were calculated. To explore if there were statistically significant differences between the specimens of the different CAD/CAM groups and the control groups, a one-way Welch’s ANOVA was conducted together with the Games-Howell post-hoc analysis.

To assess the relationship between the different mechanical properties and the fracture surface roughness, a Pearson product-moment correlation was run. The linear relationship was confirmed by an inspection of the respective scatter plots.

The significance level for statistical tests was set at α = 0.05, and α = 0.01 was set as level for high statistical significance.

## 3. Results

### 3.1. Breaking Load

**Figure 2:**
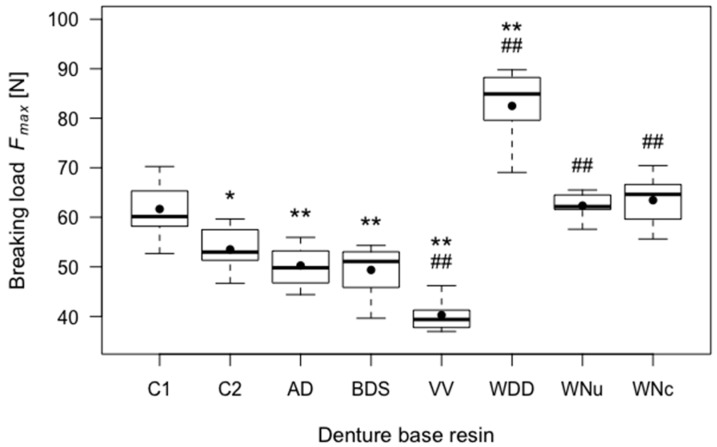
Boxplots of the breaking loads (*F_max_*) of the tested resins. Significant differences (*p* < 0.05) with respect to control group C1 are indicated by *, highly significant differences (*p* < 0.01) with respect to control group C1 are indicated by **, and ## indicates highly significant differences (*p* < 0.01) with respect to control group C2.

The heat-polymerising control group C1 had a mean breaking load of 61.66 N (SD = 5.60). The autopolymerising control group C2 had a mean breaking load of 53.51 N (SD = 4.07). The difference between C1 and C2 was statistically significant (*p* = 0.03). WDD had a statistically highly significantly increased mean breaking load in comparison to both C1 and C2 (*p* < 0.01). WNu and WNc had a significantly higher mean breaking load than C2 (*p* < 0.01), but not significantly higher than C1. AD and BDS had a significantly lower mean breaking load than C1 (*p* < 0.01), but not significantly lower than C2. Only VV had a significantly lower mean breaking load than C2 (*p* < 0.01). Therefore, the first *H*_0_ was rejected for all CAD/CAM denture groups regarding the breaking load. The results are displayed in detail in Figure 2 and Table 2.

### 3.2. Fracture Toughness

**Figure 3:**
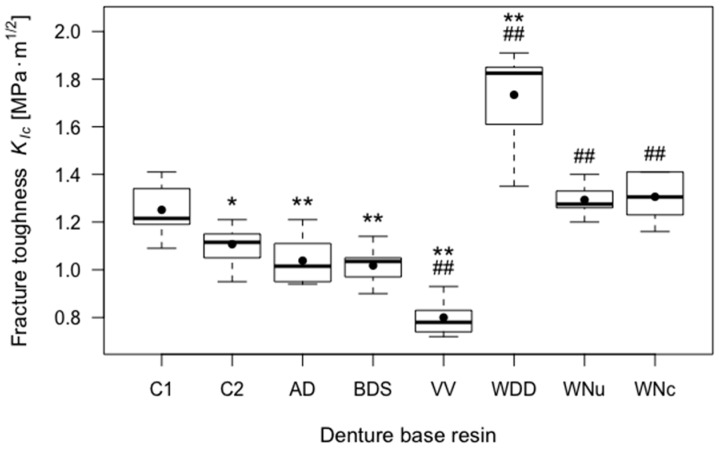
Boxplots of the fracture toughness (*K_Ic_*) of tested resins. Significant differences (*p* < 0.05) with respect to control group C1 are indicated by *, highly significant differences (*p* < 0.01) with respect to control group C1 are indicated by **, and ## indicates highly significant differences (*p* < 0.01) with respect to control group C2.

C1 had a mean fracture toughness of 1.25 MPa·m^1/2^ (SD = 0.11). C2 had a statistically significantly lower mean fracture toughness (mean = 1.11 MPa·m^1/2^, SD = 0.08) than C1 (*p* < 0.05). WDD had a significantly higher mean fracture toughness than both control groups (*p* < 0.01). WNu and WNc had a significantly higher mean fracture toughness than C2 (*p* < 0.01), but not than C1. Again, the coating did not significantly influence the cracking behaviour of the Whole You Nexteeth specimens. AD and BDS had a significantly lower mean fracture toughness than C1 (*p* < 0.01), but not significantly lower than C2. VV had a significantly lower mean fracture toughness than both C1 and C2 (*p* < 0.01). Consecutively, *H*_0_ was rejected for all CAD/CAM denture groups regarding fracture toughness. Figure 3 and Table 2 give an overview of the fracture toughness results.

### 3.3. Elastic Modulus

**Figure 4:**
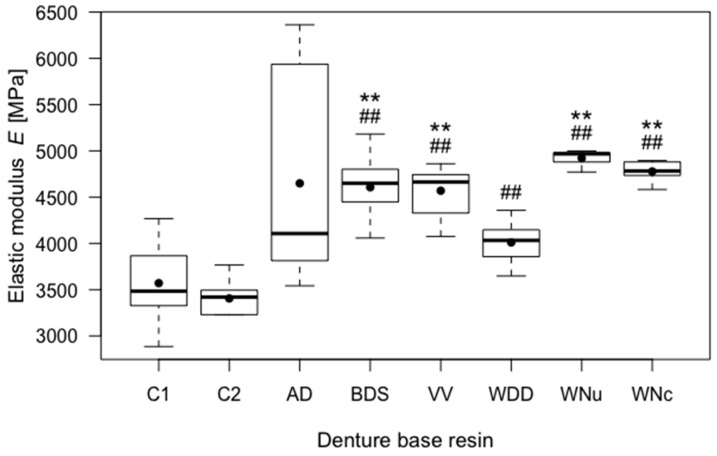
Boxplots of the elastic moduli (*E*) of the tested resins. ** indicates highly significant differences (*p* < 0.01) with respect to control group C1, ## indicates highly significant differences (*p* < 0.01) with respect to control group C2.

C1 had a rather low mean elastic modulus of 3570.24 MPa (SD = 450.75). C2 had the lowest mean elastic modulus with a value of 3405.01 MPa (SD = 178.52). The mean value difference between C1 and C2 was not statistically significant. All the CAD/CAM groups, except for AD, had statistically highly significantly increased mean elastic moduli than C2 (*p* < 0.01), and all the CAD/CAM groups, except for AD and WDD, also had highly significantly increased mean elastic moduli than C1 (*p* < 0.01). Therefore, the first *H*_0_ was rejected for all the CAD/CAM denture groups except for AD, regarding the elastic modulus. The results are fully listed in Table 2 and illustrated in Figure 4.

### 3.4. Fracture Surface Roughness

**Figure 5:**
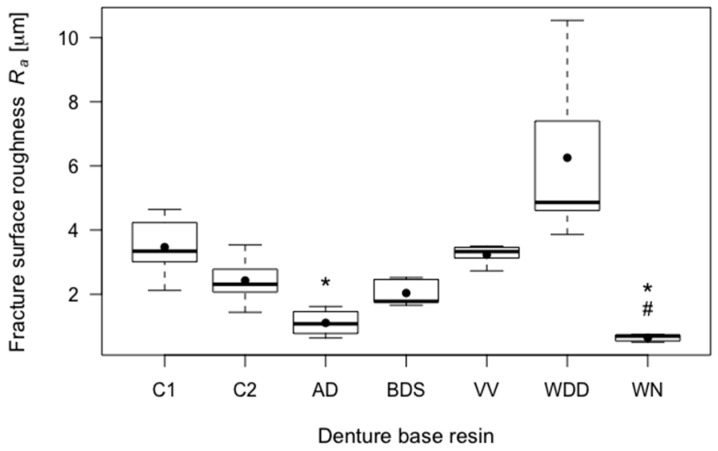
Boxplots of the fracture surface roughness (*R_a_*) of the tested resins. Significant differences (*p* < 0.05) with respect to control group C1 are indicated by *, and significant differences (*p* < 0.05) with respect to control group C2 are indicated by #.

C1 specimen fracture surfaces had a mean roughness value (*R_a_*) of 3.47 μm (SD = 0.10). C2 specimen fracture surfaces had a mean *R_a_*-value of 2.42 μm (SD = 0.79). C1 and C2 did not differ significantly in fracture surface roughness. WN specimens had by far the smoothest fracture surfaces, and the differences to both control groups were statistically significant (*p* = 0.019 for C1 and *p* = 0.050 for C2). AD specimens had a significantly smoother fracture surface than C1 (*p* = 0.032), but not than C2. WDD had rougher fracture surfaces than both control groups, but the differences were not statistically significant. Because of these results, the second *H*_0_ was rejected for the CAD/CAM denture groups WN and AD. The fracture surface results are shown in Figure 5 and specified in Table 2.

### 3.5. Statistical Correlation between Mechanical Behaviour and Fracture Surface Roughness

There were positive correlations with medium strength between the fracture surface roughness *R_a_* and the breaking load *F_max_* (r = 0.390), as well as between the fracture surface roughness *R_a_* and the fracture toughness *K_Ic_* (r = 0.344). Both correlations were statistically significant. Additionally, there was a negative correlation with medium strength between the fracture surface roughness *R_a_* and the elastic modulus *E* (r = −0.395). This negative correlation was also statistically significant. Since all correlations were statistically significant (*p* = 0.019 for the breaking load, *p* = 0.040 for the fracture toughness, and *p* = 0.017 for the elastic modulus), the third *H*_0_ was also rejected.

### 3.6. SEM Imaging of Fracture Surfaces

**Figure 6:**
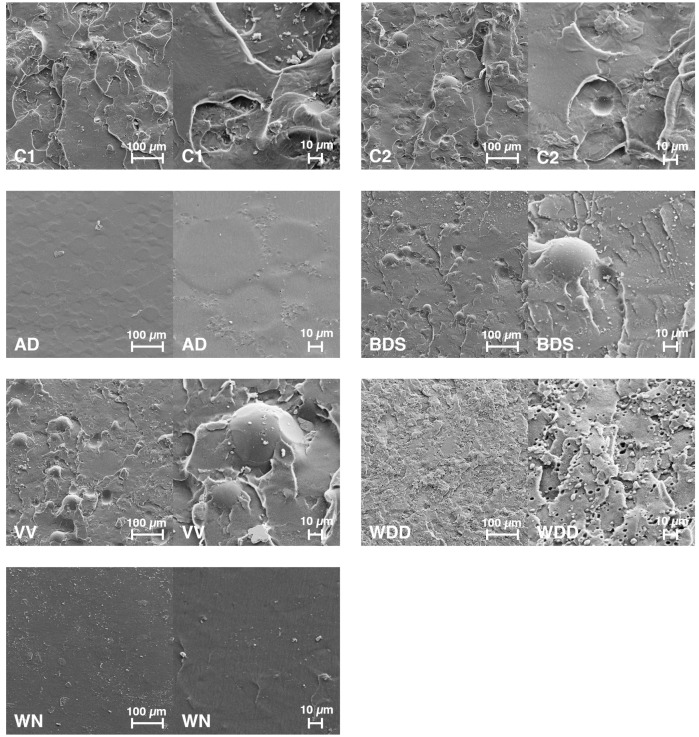
Scanning electron microscope (SEM) imaging of fracture surfaces. Abbreviations: C1: control group 1 (heat-polymerising resin); C2: control group 2 (autopolymerising resin); AD: AvaDent; BDS: Baltic Denture System; VV: Vita Vionic; WDD: Wieland Digital Dentures; WN: Whole You Nexteeth.

Figure 6 shows the SEM images taken from the different resins’ fracture surfaces.

## 4. Discussion

### 4.1. Experimental Setup

Although the sample size and the experimental setup for the three-point bending test were derived from the ISO 20795-1:2013 [20], the aim of the present study was not to assess the resins’ ISO compliance, but to compare the mechanical performances of CAD/CAM denture base resins with conventionally processed resins, particularly since previous studies investigating the mechanical properties of denture base resins had criticised the rationale of the cut-off values given in the ISO standard [5,22,23]. For calculating the elastic modulus, the ISO holds another protocol, but the formula could be adopted to enable the usage of the data obtained from the present experiments. Therefore, the comparison of the obtained values for the elastic modulus must be regarded with some caution.

For the present study, the reproducibility of the experimental protocol was verified in the preliminary experiments, in which two different examiners used two different material testing machines of the same kind.

Although generally not recommended, self-curing resin is unfortunately still often used for complete denture fabrication or relining. It was therefore included in this study. 

### 4.2. Study Findings

The breaking load quantifies the resistance of a material against fracture. In a clinical study among full denture wearers, bite forces were highest in the molar and premolar area, where they did not exceed 55 N [24]. In complete denture wearers with strong resorption of the mandibular alveolar bone, the maximal bite force was reduced to 40 N [24]. In the present study, all denture base resins exhibited breaking loads above 40 N. In a collection of 54 CAD/CAM and conventional dentures produced for material testing for our department, the mean denture base thickness varied between 1.3 mm and 3.6 mm in the palatal plate region. The specimens submitted to the three-point bending tests in the present study had a residual thickness of 5.0 mm after the pre-crack was applied. Nevertheless, the study sample geometry and the geometry of entures are completely different, not only regarding thickness. In addition, dentures in clinical use are loaded in far more complex ways since they are submitted to different types of forces impinging in different directions. For a valid prediction of the clinical stress resistance, other methods, such as finite element analysis, will be necessary. However, the results of the present study definitely show that none of the tested CAD/CAM resins, except for VV, cracked at lower breaking loads than the well-used autopolymerising resin. It may therefore be assumed that CAD/CAM dentures will not have an increased fracture rate, provided the usual denture base thickness is maintained. Lower denture base thicknesses, however, must be regarded with caution. 

The fracture toughness describes the ability of a specimen containing a predetermined breaking point to resist failure. For complete dentures, this damage tolerance is highly relevant, as it reflects the denture’s ability to remain intact even with a beginning crack, up until the denture can be repaired. In the present study, two groups (WDD and WN) had significantly higher fracture toughness than the control groups, indicating that crack growth was slower in these groups. The differences between the best and the poorest performance were over 100%, which is why it may be recommendable to refrain from producing CAD/CAM dentures with only minimum material thicknesses.

The elastic modulus is another parameter with high clinical relevance for complete dentures, as denture base materials with high elastic moduli are more resistant against elastic deformation and therefore allow the fabrication of dentures with thinner bases. The elastic moduli of all CAD/CAM denture base resins examined in the present study were higher not only than in our control groups, but also than in other reports investigating conventionally processed denture base materials [2,5]. At the same time, they also laid above the reference values for pure PMMA [25]. Thus, the enhanced elastic moduli might be attributable to additives. Another recent study similar to ours also found that a CAD/CAM manufactured denture base resin had a significantly higher flexural modulus than heat-cured PMMA, and the author related this finding to a low concentration of residual MMA monomer in the CAD/CAM denture resin [26].

To our knowledge, the present study is the first to examine the fracture surface roughness of denture base resins. Therefore, our assumptions about which structural components could cause the different surface patterns are, in part, speculative. It is, however, not conclusive to assume that the fracture surface roughness should be influenced by the polymer chain length, since the intramolecular forces do not increase with growing chain length. A far more plausible cause of inhomogeneity within the micro-structure is the polymer powder geometry, size, and distribution. According to Huggett et al. [7], denture base resins contain essentially three phases: a polymer powder unpenetrated by monomer, a (residual) monomer matrix with the inter-penetrating network, and a compound phase of polymer powder infiltrated by a monomer liquid [27]. Huggett plausibly deduced that an increased imbibition of powder particles by the monomer liquid would lead to a deeper inter-penetrating network and also increase the microstructure’s homogeneity [7].

Another interesting finding was the interrelation between the different mechanical properties and between the mechanical properties and the fracture surface texture. WDD showed the highest breaking load and the highest fracture toughness, but at the same time showed a lower elastic modulus. The explanation for this inverse relation might be that the resin possibly contains plasticising agents, which have been shown to increase the impact strength while reducing the material’s elastic modulus [28]. Besides a higher breaking load, WDD also had the roughest fracture surface. The groups AD and BDS, on the other hand, were on the opposite side of the mechanical property ranges. These groups had a rather low breaking load and low fracture toughness and, at the same time, a high elastic modulus (material stiffness) and smooth fracture surfaces. C1 and C2, the heat- and autopolymerising resin groups, showed an intermediate breaking load and fracture toughness, but the lowest elastic modulus. The fracture surface roughness, again, was within the middle range of the observed spectrum. WN resin, on the other hand, exhibited a completely different combination of mechanical properties. While WN specimens showed intermediate values for breaking load and fracture toughness, they also showed the highest material stiffness, and all specimens within this group had extremely smooth fracture surfaces.

The SEM images (Figure 6) nicely illustrate the differences in fracture surface textures between the various resins. The WN and AD specimen fracture surfaces present with very even and smooth surfaces without visible particles. BDS, VV, C1, and C2 surfaces are visibly rougher and seem to be streaked with coarse clods of different size. In addition, the fractures reveal spherical particles with diameters of around 40 μm, probably PMMA microspheres in BDS, VV, and C2 specimens, with apparently different microsphere content. BDS resin seems to have the highest microsphere content, followed by VV and then C2. WDD resin, on the other hand, presents a rather fine-granular fracture surface texture, and at a higher magnification seems to be interfused with small oval cavities, which might either represent micro-pores or perhaps residues of soluble particles washed out during the purifying procedure (application of residue-free aerosol) necessary for preparing the specimens for SEM imaging.

## 5. Conclusions

The results of the present study demonstrate that there are large variations in fracture resistance between the different available CAD/CAM denture base resins. The results also show that CAD/CAM denture base resins do not generally have a higher fracture tolerance or a higher resistance against crack growth than manually processed auto- or heat-polymerising resins. The customary coating on Whole You Nexteeth dentures does not seem to have a significant influence on the crack or fracture resistance. All CAD/CAM denture base resins seem to have higher elastic moduli than heat- or autopolymerising resins, and the fracture surface analyses indicate that the resin’s microstructure might be responsible for the different mechanical properties, rather than the polymer chain length, which would be influenced by the curing protocol. We therefore assume that the differences regarding the mechanical properties may be attributed to the resin’s composition rather than the industrial processing. Against this background, however, the advertised lower minimum thicknesses of CAD/CAM denture bases should be regarded with some caution.

## Figures and Tables

**Table 1:** Study groups and manufacturers.

Group	Abbreviation	Product Name	Manufacturer	Head Office
1	AD	AvaDent Digital Dentures	Global Dental Science Europe BV	Tilburg, Netherlands
2	BDS	Baltic Denture System	Merz Dental GmbH	Lütjenburg, Germany
3	VV	Vita VIONIC	Vita Zahnfabrik	Bad Säckingen, Germany
4	WDD	Wieland Digital Dentures	Wieland Dental + Technik GmbH & Co. KG Ivocar Vivadent AG	Pforzheim, Germany/Schaan, Liechtenstein
5	WNu	Whole You Nexteeth (uncoated)	Whole You Inc.	San Jose, US
6	WNc	Whole You Nexteeth (coated)	Whole You Inc.	San Jose, US
7	C1 (control group 1)	Candulor Aesthetic Red (heat-curing resin)	Candulor AG	Glattpark, Germany
8	C2 (control group 2)	Candulor Aesthetic Blue (self-curing resin)	Candulor AG	Glattpark, Germany

**Table 2:** Detailed mechanical properties and fracture surface roughness.

Parameter	Group	Mean	SD ^a^	*p* (Compared to C1)	*p* (Compared to C2)
Breaking load *F_max_* (N)	C1	61.66	5.60	-	0.030
	C2	53.51	4.07	0.030	-
	AD	50.26	4.02	0.002	0.629
	BDS	49.37	4.91	0.001	0.448
	VV	40.27	3.40	0.000	0.000
	WDD	82.49	7.47	0.000	0.000
	WNu	62.35	2.44	1.000	0.001
	WNc	63.44	4.91	0.993	0.002
Fracture toughness *K_Ic_* (MPa·m^1/2^)	C1	1.25	0.11	-	0.049
	C2	1.11	0.08	0.049	-
	AD	1.04	0.10	0.004	0.644
	BDS	1.02	0.07	0.001	0.170
	VV	0.80	0.07	0.000	0.000
	WDD	1.73	0.19	0.000	0.000
	WNu	1.29	0.6	0.947	0.000
	WNc	1.31	0.09	0.913	0.001
Elastic modulus *E* (MPa)	C1	3570.24	450.75	-	0.950
	C2	3405.01	178.52	0.950	-
	AD	4649.15	1110.93	0.171	0.077
	BDS	4606.38	325.93	0.000	0.000
	VV	4569.16	267.40	0.001	0.000
	WDD	4009.95	200.00	0.175	0.000
	WNu	4921.05	87.85	0.000	0.000
	WNc	4777.01	110.72	0.000	0.000
Fracture surface roughness *R_a_* (µm)	C1	3.47	0.10	-	0.568
	C2	2.42	0.79	0.568	-
	AD	1.11	0.38	0.032	0.118
	BDS	2.04	0.42	0.197	0.942
	VV	3.23	0.31	1.000	0.405
	WDD	6.25	2.74	0.447	0.205
	WN	0.65	0.11	0.019	0.050

^a^ Standard deviation.
